# Perceptions of Clinical Connectedness Among Hospital Environmental Service Workers

**DOI:** 10.1001/jamanetworkopen.2024.53775

**Published:** 2025-01-13

**Authors:** Nicholas Allis, Zhi Chen, Leah G. Jones, Timothy Kohanski, Zane Suttmore, Samantha Turnquest, Joyce Appiah-Asare, Stephen Appiah-Asare, Kendell Battle, Terry Frayer, Fateen Gilkey, Sherry D. Jones, Kelvin Little, Susan Murphy, Michelle Robinson, Anita Rouse, Jason Rupert, Moustapha Salawu, Zoreslava Osiv, Scott Rosas, Telisa Stewart

**Affiliations:** 1 Department of Public Health and Preventive Medicine, State University New York (SUNY) Upstate Medical University, Syracuse, New York; 2 Department of Environmental Services, SUNY Upstate Medical University, Syracuse, New York

## Abstract

**Importance:**

Environmental service workers (ESWs) have a critical role within the hospital infrastructure and are at the frontline of infection prevention. ESWs are highly trained in managing all forms of regulated waste, which includes biohazardous waste, and are responsible for the overall patient experience, janitorial work, and infection prevention. Without environmental services, patients have a 6 times greater risk of being infected by pathogens from patients who previously occupied their room.

**Objective:**

To understand how ESWs felt proud, connected, and not connected to their hospital-based clinical teams.

**Design, Setting, and Participants:**

This qualitative study included 10 ESWs who were members of clinical care teams at a medical university hospital in upstate New York. Data were collected from February to May 2024.

**Main Outcome and Measures:**

Participants were asked to take pictures and write vignettes about what makes them feel proud of their work, how they feel connected to their clinical team, and how they feel disconnected from their clinical team.

**Results:**

This study included 10 participants (5 males [50%]; 10 non-Hispanic Black or African American individuals [100%]; mean [range] age, 53 [38-66] years; 8 were high school graduates or had some college credit [80%]). When asked about their feeling of pride, participants expressed maintaining a clean space, having meaningful relationships, and helping others as generating a sense of pride. Participants’ feelings of disconnectedness were felt by their colleagues not following the rules set in place. In addition, participants reported feeling invisible, not listened to, unappreciated, and undervalued. Participants felt connected to their clinical teams by feeling a sense of community, having valuable relationships, and being able to communicate with members of the clinical team. Additionally, participants expressed a collective desire to be acknowledged, recognized, and treated as equals on the clinical team.

**Conclusions and Relevance:**

The study found that ESWs identified feeling both connected to and disconnected from their clinical teams. Based on these results, hospital infrastructure and leadership should continue to exhaust all efforts to explore work experiences of ESWs to improve job retention, morale, satisfaction, overall clinical teamwork, and comradery.

## Key Points

**Question:**

How do environmental service workers (ESWs) feel proud, connected, and not connected to their clinical teams?

**Findings:**

In this qualitative study of 10 participants, ESWs felt proud and felt connected to their clinical teams, but also felt not connected, undervalued, and underappreciated.

**Meaning:**

These findings suggest that hospital leadership should exhaust all efforts to explore ESWs’ work experiences to improve job retention, morale, satisfaction, overall clinical teamwork, and comradery.

## Teaser

This cohort study examines how environmental service workers felt proud, connected, and not connected to their hospital-based clinical teams.

## Introduction

In the US, there are more than 81 000 janitors and cleaners who work in hospitals.^1^ These environmental service workers (ESWs) are an integral part of the clinical care team and perform mission-critical hospital functions.^2,3^ The health care industry, in particular hospitals, are highly regulated and scrutinized by enforcement agencies. ESWs substantially contribute to the ongoing regulatory compliance within hospital infrastructure.^2^ ESWs are highly trained in managing all forms of regulated waste, which includes biohazardous waste, and responsible for the overall patient experience, janitorial work, and infection prevention.^2,4,5,6^

Within the hospital setting, ESW are the frontline of infection prevention and are continuously exposed to viruses, pathogens, and bacteria when preparing and maintaining patient rooms.^7,8^ On average, there are 1.7 million health care–associated infections (HAIs) that contribute to almost 100 000 associated deaths each year.^7^ Without environmental services, patients have a 6 times greater risk of being infected by pathogens from previous patients who once occupied their room.^8,9^

Currently, there is clear and direct research emphasizing the importance of the roles of ESWs and their critical contribution to the overall health, safety, and well-being of patients and clinicians.^2,3,8,9,10,11,12,13^ Despite this, they continue to be underappreciated, underpaid, understaffed, unrecognized, and viewed as easily replaced unskilled laborers.^7,8,14,15^ Research has shown that ESWs’ experiences and feelings are directly associated with the effectiveness of their efforts and the quality of care for patients.^11,16^ However, more research needs to focus on the lived experiences, feelings, and perspectives of ESWs to help improve job retention, job satisfaction, functionality, communication, patient satisfaction, clinical care teamwork, and health care outcomes.

Qualitative methods historically have been used to help demonstrate feelings and experiences of participants.^17,18,19^ In particular, the photovoice technique has been well established in the literature for more than 30 years and was used to better understand the lived experiences of unheard, overlooked, and marginalized populations.^20,21,22,23,24,25,26,27,28,29^ As part of the methods, participants were asked to express their thoughts, feelings, and emotions through pictures and vignettes.^22,24,30,31,32^ Participants were asked to work individually to capture images in response to 1 or more prompts and discuss their findings collaboratively throughout the process. This method allowed for recording and reflecting areas of growth and promoting critical dialogue about important issues. Pictures and vignettes were recorded and shared with decision-makers to foster social change.

To our knowledge, this study was the first quality improvement (QI) project to document the lived experiences of ESWs using a photovoice technique. The project sought to understand how ESWs felt proud, connected, and not connected to their hospital-based clinical teams in the context of the role in the clinical setting.

## Methods

### Study Design and Participants

This qualitative study was approved by the institutional review board at SUNY Upstate, which determined that this study did not meet the definition of human participant research and documentation of consent was not required. This study followed the Consolidated Criteria for Reporting Qualitative Research (COREQ) reporting guideline.

This qualitative study used the photovoice technique and took place at a large academic medical school in upstate New York. The project took place from February to May 2024. The QI team used a modified community-based participatory research (CBPR) approach, which used a partnership approach that equitably distributed the project’s responsibilities and ownership.^33,34^ This CBPR project identified the ESWs as an underserved and disadvantaged community. In viewing the ESWs as a group of individuals with a common problem and shared interest, the project sought to strengthen the community through engagement and empowerment.^33,34^ The QI team leveraged their relationships with the environmental services department to develop the study and partnered with the department’s director to develop the protocol. The materials were vetted by hospital leadership and by a random sample of ESWs; minor adjustments were made to the study questions. Next, the environmental services director recruited a convenient sample of 10 ESWs who worked directly with clinical teams to participate on the project; 10 is an ideal sample size for the photovoice method.^35,36,37^

The study participants self-reported sex, race, and ethnicity categories, which were derived from the National Center for Health Statistics. Self-reported variables were important in describing the participants and may influence the generalizability of the results.^38^ The analytical sample was only restricted by employment role; only ESWs were eligible to participate.

### Study Procedure and Data Collection

The QI team collaborated with the ESW department to create the procedures specific to virtual meetings and a condensed timeline. There was a total of 10 virtual meetings that were 1 hour in length, and participants engaged using a video conferencing platform. Participants were asked to take a minimum of 2 and a maximum of 5 pictures in response to 3 prompts: (1) what makes them feel proud of their work; (2) how they feel connected to their clinical team; and (3) how they feel disconnected from their clinical team. Pictures were uploaded to a shared folder. Participants met twice a week for 5 weeks (eTable in Supplement 1). The first and second sessions reviewed the project goals, photography skills, guidelines, and ethical considerations. Thereafter, each week a topic was presented during the session, and participants reviewed the photos taken, experiences, and commonalities in the following session. After the discussion, each participant was paired with a team member (faculty and lead experts: T.S., S.R.; administrative assistant: Z.O.; students: N.A., Z.C., L.J., T.K., Z.S., S.T.) to select their best photo and drafted their vignette using the SHOWED method (What do you see here? What is really happening here? How does this relate to our lives? Why are things this way? How could this image educate people and what can we do about it?). The SHOWED method is an evidence-based technique that generates information by asking participants about their lived experiences.^24,26,27,39,40,41,42^

The team member typed what the participant said verbatim. Participants were prompted using the traditional SHOWED questions for the not connected prompt. The SHOWED method was adjusted to fit prompts of proud and connected by slightly altering the SHOWED method. Specifically, the last 2 SHOWED questions were altered by asking participants, “How could this image educate people and what does this picture share about you?” and “What can I do to continue feeling this way?”

A photovoice community event was held on campus to showcase the pictures and vignettes of the participants on May 2, 2024. The event took place at a central location inside the hospital and was open to academic faculty, hospital employees, students, and family members. Each participant had their 3 selected photos and corresponding vignettes printed on posters and displayed on easels for all to see. Leaders, decision-makers, and hospital staff from across the institution were invited; decision-makers had robust discussions with the ESWs about concerns identified and celebrated the successes. After the event, meetings were held to discuss the concerns and implement hospital quality improvement. Changes included, but were not limited to, systematic adjustments, environmental changes, and discussions about how to improve culture.

### Data Analysis

A thematic analysis was conducted. The vignettes were qualitatively coded using ATLAS.ti version 24 (Lumivero) to identify themes; photo image analysis was not conducted because the interpretation provided by the vignettes provided more insight.^43,44,45,46,47,48^ Each topic was assigned to 2 student team members, and the teams created and tested their respective codebooks. The coders were new to the field of qualitative analysis and had no prior experience with the field of environmental services. The first coder in each pair initiated analysis by identifying representative quotes within the vignettes and assigning 2 codes—the question being answered and the theme code from the corresponding question that they thought best applied to the quote. Once the first coder was done, they created a duplicate file and stripped the theme codes. The second coder then independently assigned theme codes to the previously identified quotes and corresponding questions. Once the coding was complete, an interrater agreement was run. Coders discussed and resolved any disputes in coding until an interrater agreement of 100% was reached.^49,50,51^ From there, emerging themes were identified.^52^ The project used information power to guide the analysis. The sample size of the project was continuously assessed during the development and analysis. The sample size was deemed appropriate for informing new findings and addressing the study aims. The data generated was in-depth enough to provide insight into ESWs’ perceptions of pride and feelings of being connected and not connected to the clinical team.^53^

## Results

This study included 10 participants (5 males [50%]; 10 non-Hispanic Black or African American individuals [100%]; mean [range] age, 53 [38-66] years) (Table 1). Six individuals (60%) were high school graduates or equivalent, 2 individuals (20%) had some college credit but no degree, and 2 individuals (20%) had less than a high school diploma. Two individuals (20%) had been in the environmental services department for 5 years or less, 3 individuals (30%) for 6 to 10 years, 1 individual (10%) for 11 to 15 years, 1 individual (10%) for 16 to 20 years, and 3 individuals (30%) for 21 years or more. .

**Table 1.** Participant Demographics

Demographic	Participants, No. (%)
Sex	
Male	5 (50)
Female	5 (50)
Race	
American Indian	0
Asian	0
Black or African American	10 (100)
Native Hawaiian or Other Pacific Islander	0
White	0
Other	0
Age, y	
Mininum	38
Maximum	66
Ethnicity	
Hispanic	0
Non-Hispanic	10 (100)
Education level	
Less than a high school diploma	2 (20)
High school graduate or equivalent	6 (60)
Some college credit but no degree	2 (20)
Years in EVS, y	
0-5	2 (20)
6-10	3 (30)
11-15	1 (10)
16-20	1 (10)
≥21	3 (30)

Abbreviation: EVS, environmental services.

### Proud

When asked what made them feel proud, participants expressed maintaining a clean space within their assigned areas of the hospital. Additionally, participants described feeling proud through the meaningful relationships they developed within their hospital community and about helping others in their assigned areas of the hospital campus.

When asked how their work makes them feel, participants expressed feeling motivated to do a good job, and participants described a sense of gratification with completing their work. When asked how they could continue feeling proud, participants described the need to receive more acknowledgment of their work. Participants also expressed being diligent with their job responsibilities as a source of pride. Additionally, participants expressed the desire to continue supporting others in the clinical community as well as receiving that same support from their fellow clinical care team members (Table 2).

**Table 2.** Questions and Themes for Feeling Proud

Questions and themes	Example (participant identification No.)
What makes you feel proud?	
Clean space	“I step out of my usual daily responsibilities to clean and disinfect the board. I like to keep it clean, so they don’t get sick from touching something.” (3)
Community	“I really feel honored that being an EVS worker gives me the opportunity to create connections with all types of people.” (2)
Helping others	“The hospital is for the patient; cleanliness is a part of taking care of the patient. When the environment is clean, it helps everybody. Patients are the most important people.” (5)
How does it make you feel?	
Motivated	“I’m not lazy—no job is too small or too big for me to handle.” Participant (6)
Gratified	“When I first started, I wasn’t giving it my all and I thought there would be no way I could master it. Now I can show others how to use it and I am proud.” (10)
How do we continue feeling proud?	
Acknowledgment	“Being appreciated for the effort I put into that work and the what I do in general would keep me feeling proud, as it motivates me and it is very inspiring to be appreciated for my hard work.” (1)
Diligence	“While I am here, I try to do my best. While on the clock, I am giving everything I got. When you punch in, you must love what you do. The time clock is how I communicate my commitment and pride in my work.” (7)
Support	“We can gather in a clean space and that really makes me proud of the job that I do here. I am honored to be a part of a group that keeps this hospital moving.” (9)

Abbreviation: EVS, environmental services.

### Not Connected to the Clinical Team

When asked what makes them feel not connected to the clinical team, participants expressed feeling not connected when members of the clinical team did not follow the rules set in place. Participants described being invisible due to being ignored, not listened to, unappreciated, and undervalued. When asked how it made them feel, participants expressed feeling disrespected by the clinical community. When asked what the solution was to feeling not connected, participants expressed the need for better communication from the clinical community (Table 3).

**Table 3.** Questions and Themes for Feeling Not Connected

Questions and themes	Example (participant identification No.)
How does this make me feel?	
Disrespected	“They don’t necessarily see you as a person. They see you for what you do. But I am not defined by my job as a cleaner.” (8)
What makes me not connected?	
Not following the rules	“This has been going on for a long time. Years. It says on top of the linen cart to not overload it. They just disregard it and not care.” (4)
Invisibility	“This kind of shows the amount of work I do. People don’t understand the amount of work we do on a daily basis to make things as clean as possible for them.” (8)
What is the solution?	
Communication	“I always say communication can help address this disconnect, communication is the key.” (9)

### Connected to the Clinical Team

When asked what made them feel connected to the clinical team, participants described a sense of community and highlighted the desire of creating valuable relationships with members of the clinical team. Participants described being able to easily communicate with members of the clinical community. When asked how feeling connected to the clinical team made them feel, participants described feeling united with the clinical community and highlighted the importance of working as 1 team. Additionally, participants described feeling happy, especially when the clinical community goes out of their way to help the environmental services employees. When asked how they could keep feeling connected, participants expressed the desire to have more support from the clinical community by having them go beyond their normal responsibilities. Participants also described the desire to have a friendlier relationship with the clinical community and being able to talk with other clinical staff members (Table 4).

**Table 4.** Questions and Themes for Feeling Connected

Questions and themes	Example (participant identification No.)
What makes me feel connected?	
Positive coworker relationships	“When you work in an environment full of nice, kind people, who are appreciative and have smiles on their faces, or are at least friendly, it makes you want to continue to go on and on with your work, as well as give it your all.” (1)
Communication	“Sometimes when they finish up early in the rooms and are done with clinic for the day, they give me a call so I can come in early to prep for the next day.” (9)
How does that make me feel?	
United	“There is a feeling that no one is bigger than the next person. We all have to work as one.” (6)
Happiness	“They call me directly and that’s why I love this floor.” (3)
How can we keep feeling connected?	
Beyond	“Cuz they see I’m backed up and they’re helping me out. That little bit helps a lot.” (4)
Talking	“I would like if the clinical team would check in on me…When I say hello, I would like people to respond instead of ignoring me.” (3)

## Discussion

From the CBPR perspective, the project was successful in creating a collaborative photovoice project.^22,34,54^ ESWs provided tangible evidence about their deep sense of identity and their commitment to their profession through the photos and vignettes (eAppendix 2 in Supplement 1). The study provided a platform for ESWs, public health, and hospital leadership to co-learn about the unique experiences and perspectives of the ESW team.^33,34^ This study fostered social capital by building on the strengths of their community.^54^ The community dissemination event was the pinnacle moment where ESWs and leadership exchanged information and established sustainable solutions to address some of the concerns.

The data analysis suggested that regardless of the topic, some overarching positive ideas emerged. There was a consistent sense of pride that resonated throughout all participant’s vignettes which allowed us to identify their work ethic, pride, diligence, and sense of community. This feeling of pride appeared to be attributed to both intrinsic and extrinsic factors. For example, when it came to intrinsic factors, helping others, building connections with fellow clinical team members, and being diligent in their work emerged. Extrinsically, when others recognize their hard work and acknowledge it, it brings the ESWs a sense of pride in their role on the clinical team. To support ESWs, hospital leadership should consider implementing a reward or recognition process to further promote pride and celebration among personnel and in the institution.

Areas for improvement revealed disrespect was profoundly perceived. There appeared to be a pervasive feeling of frustration stemming from disrespect, lack of communication, not following the rules, and feeling invisible to the clinical care teams. This highlights the system and organizational hierarchy issues and problems with a lack of humility that was able to be explored by this study. These areas can be improved by personnel within the system by self-reflecting and self-critiquing the hierarchical issues.

For ESWs to continue feeling connected to their clinical teams, there was a common consensus that improving communication between the ESWs and the clinical community could serve as an effective solution. Open discussions prompted by leadership during department meetings could help improve and maintain communication.

Additionally, participants expressed a collective desire to be acknowledged, recognized, and be treated as equal individuals on the clinical team. Teams perceived to be successfully connected used acknowledgment, appreciation, and recognition as tools for improved connectedness. These teams also had a higher level of cultural humility and engaged in friendly and meaningful interactions.

Current research is limited about ESWs. It has been noted that ESWs have a long-standing respect for humanity, a duty to help protect patients and families from disease, and a practice of connecting with patients.^55,56^

Additionally, their feelings and attitudes may impact their intent to clean.^16^ However, to our knowledge, there is no research regarding their perceptions of connectedness to their clinical teams. This study adds to the literature about the perceptions of clinical connectedness through photographs.

Patient satisfaction and patient outcomes are associated with ESW morale and attitudes because of ESW’s role in minimizing exposure to HAIs, viruses, bacteria, and pathogens.^3,4,5,6,7,8,9,10,11,12,13,14^ Being the primary defenders against biohazardous waste, pathogens, and HAIs for patients and hospital personnel, ESWs should be celebrated, recognized, and treated with the utmost respect and care; however, that is not the case.^2^ They are consistently and unequivocally undervalued, understaffed, overlooked, and underappreciated by their fellow clinical team members and hospital leadership.^2,12^ This study suggests the need to have further research and commitment focused on the feelings of ESWs and how to better serve the profession. Hospital leadership should exhaust all efforts to protect their ESWs against exposure to pathogens, prevent burnout and feeling belittled to increase the safety and well-being of ESWs, retention rate, and patient outcomes.^2,8,11,57^

### Limitations

This study has limitations. This study was limited to English-speaking participants who self-identified as Black or African American. Although deemed sufficient for this study, a larger sample size with more diversity may have helped us gain additional perspectives and experiences. There is also the limitation of what participants were allowed to take photographs of due to the Health Insurance Portability and Accountability Act and consideration to maintain anonymity of the clinical team composition. Analysis of the vignettes and determinations of themes are suspectable to subjective interpretation. The coders consisted of student analysts who were new to the field of qualitative research, and the subjectivity was limited by using 2 coders, conducting the interrater agreement, and ensuring 100% agreement. This study has modified CBPR method because not all aspects were used due to hospital infrastructure and time limitations.

## Conclusions

In this qualitative study, ESWs revealed feeling both connected and not connected to the clinical teams. This is represented by their feelings of being disrespected, being invisible, needing acknowledgment, and wanting a stronger sense of comradery among the clinical team. Additional efforts should focus on understanding the lived experiences of ESWs and improving ESWs feeling of connectedness and experiences with the clinical teams. Based on these results, hospital infrastructure and leadership should continue to exhaust all efforts to explore ESWs work experiences to improve job retention, morale, satisfaction, overall clinical teamwork, and comradery. Hospital leadership should create an advisory board of ESWs and hospital decision-makers to review concerns, engage in dialogue, and to continue quality improvement. Continued qualitative research should be done to better understand ESWs work life and connectedness to their clinical teams.

## Notes

Supplement 1. **eTable. **Timeline of Project Activities **eAppendix 1.** Questions and Prompts **eAppendix 2.** Participant Photographs

Supplement 2.
Data Sharing Statement
