# Supplementary material for: Perceptions of Clinical Connectedness Among Hospital Environmental Service Workers
Source: JAMA Netw Open. 2025 Jan 13;8(1):e2453775. doi: 10.1001/jamanetworkopen.2024.53775 (PMC11731157; doi:10.1001/jamanetworkopen.2024.53775)
Supplement: Supplement 1. — eTable. Timeline of Project Activities eAppendix 1. Questions and Prompts eAppendix 2. Participant Photographs [file jamanetwopen-e2453775-s001.pdf]

Supplemental Online Content

Allis N, Chen Z, Jones L, et al. Perceptions of clinical connectedness among hospital environmental service workers. *JAMA Netw Open.* 2025;8(1):e2453775. doi:10.1001/jamanetworkopen.2024.53775

Supplement 1. eTable. Timeline of Project Activities

eAppendix 1. Questions and Prompts

eAppendix 2. Participant Photographs

This supplemental material has been provided by the authors to give readers additional information about their work.

© 2025 Allis N et al. *JAMA Network Open*.

eTable. Timeline of Project Activities

**Project activities**	**Week 1**			**Week 2**			**Week 3**			**Week 4**			**Week 5**		
	**Mon**	**Tue**	**Wed**	**Mon**	**Tue**	**Wed**	**Mon**	**Tue**	**Wed**	**Mon**	**Tue**	**Wed**	**Mon**	**Tue**	**Wed**
Project introduction, rules, and procedures	x		x												
Topic introduced and protocol reviewed				x			x			x			x		
Data collection: 48 h of photography					x			x			x			x	
Group discussion, select 1 picture, complete SHOWED method						x			x			x			x

© 2025 Allis N et al. *JAMA Network Open*.

eAppendix 1. Question and Prompts

Proud:

- What makes you feel proud?
- How does it make you feel?
- How do we continue feeling proud?

Not Connected:

- What makes me not connected?
- How does this make me feel?
- What is the solution?

Connected:

- What makes me feel connected?
- How does that make me feel?
- How can we keep feeling connected?

© 2025 Allis N et al. *JAMA Network Open*.

eAppendix 2. Participant Photographs

1. **ePhotos for Participant One**

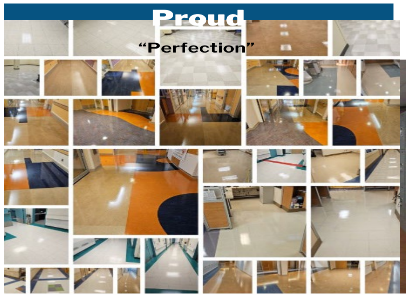

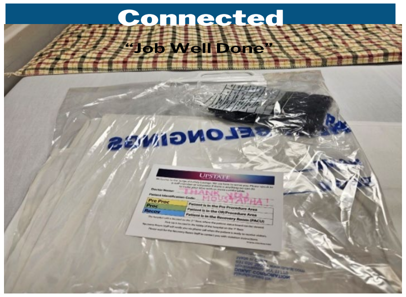

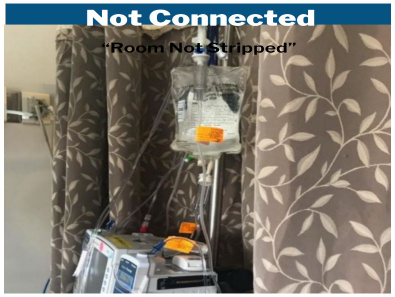

2. **ePhotos for Participant Two**

© 2025 Allis N et al. *JAMA Network Open*.

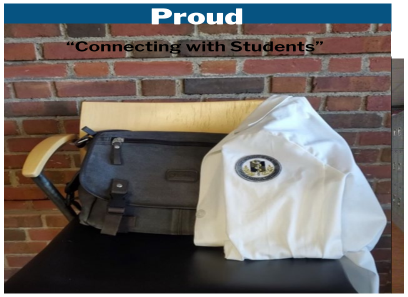

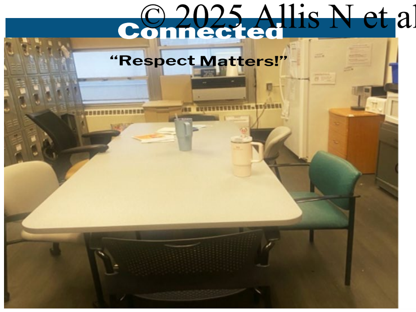

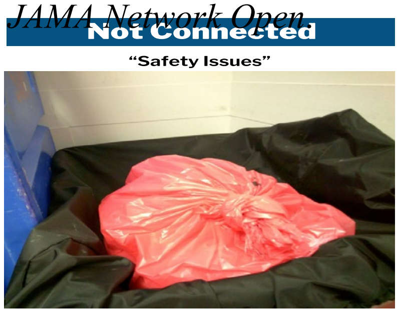

3. **ePhotos for Participant Three**

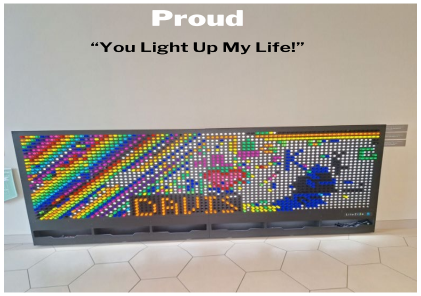

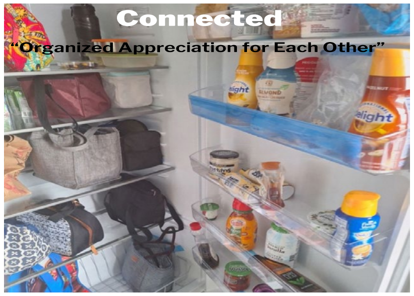

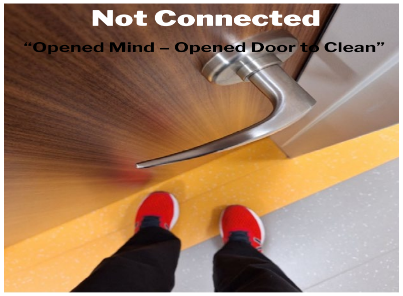

4. **ePhotos for Participant Four**

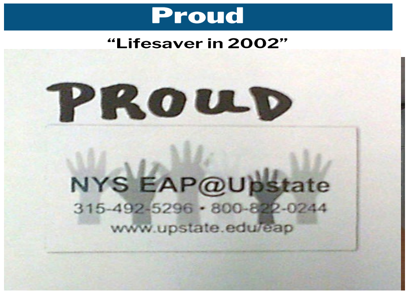

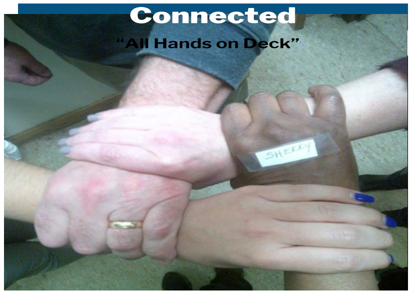

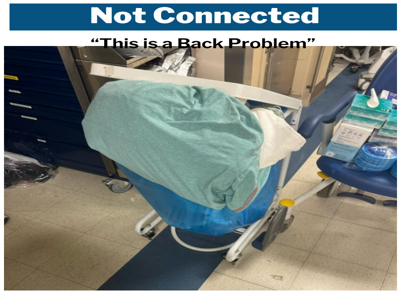

5. **ePhotos for Participant Five**

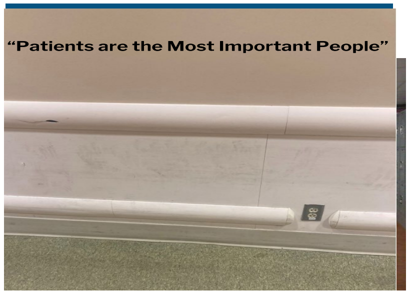

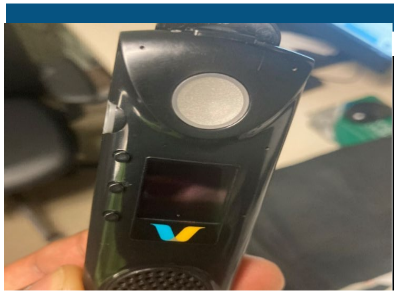

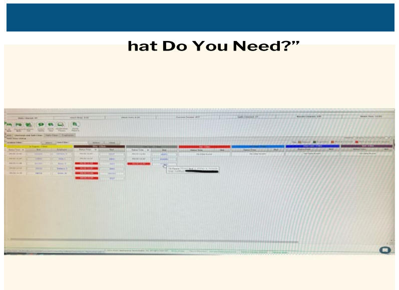

6. **ePhotos for Participant Six**

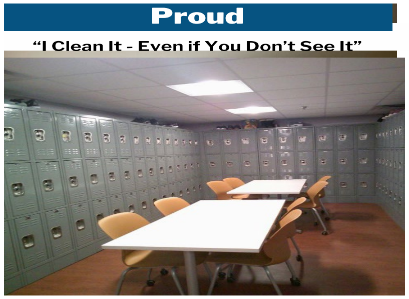

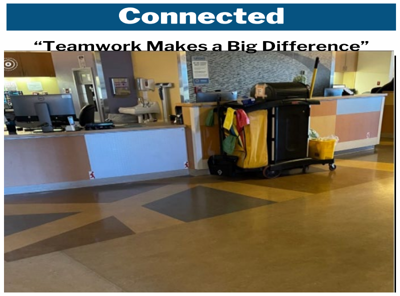

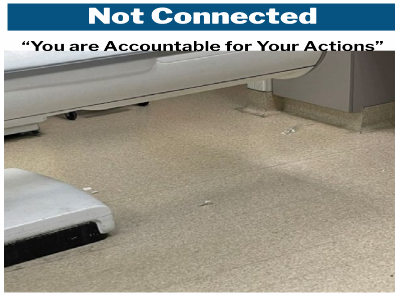

7. **ePhotos for Participant Seven**

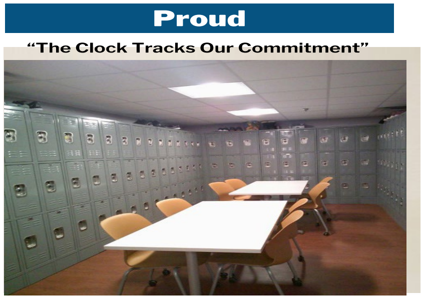

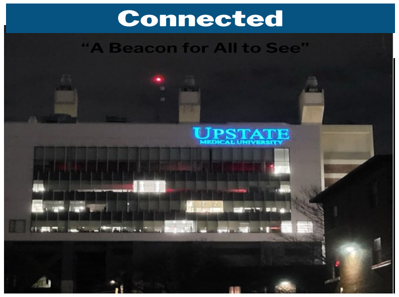

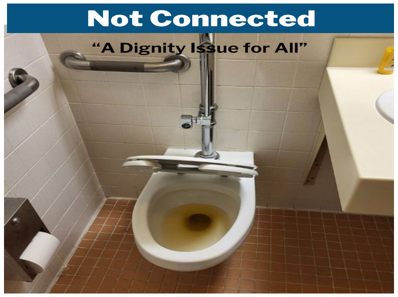

8. **ePhotos for Participant Eight**

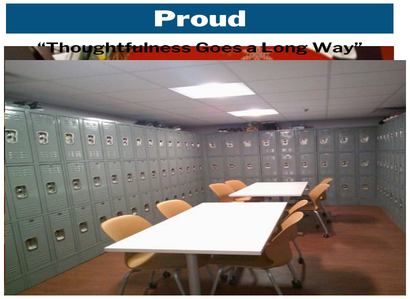

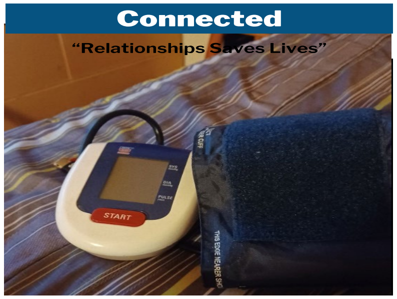

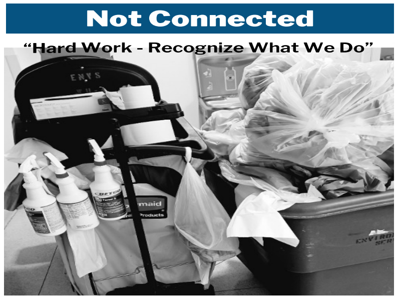

9. **ePhotos for Participant Nine**

© 2025 Allis N et al. *JAMA Network Open*.

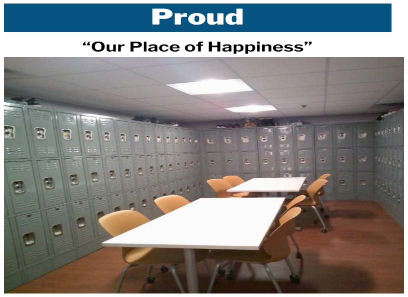

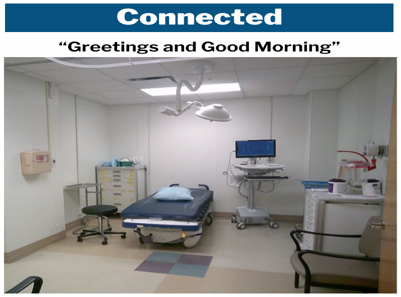

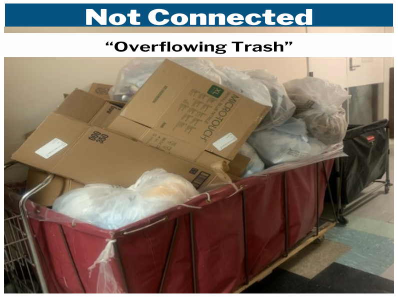

10. ePhotos for Participant Ten

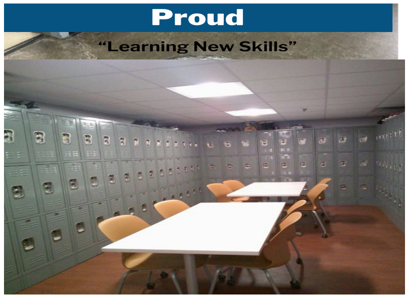

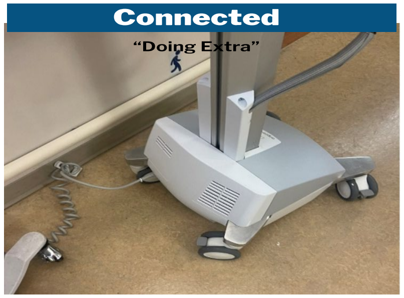

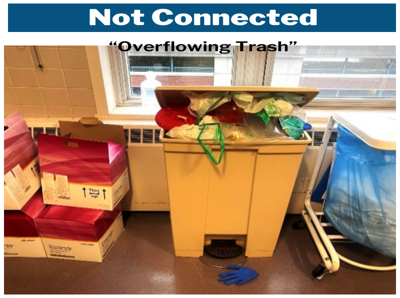

© 2025 Allis N et al. *JAMA Network Open*.
