# Supplementary material for: Perceptions of Clinical Connectedness Among Hospital Environmental Service Workers
Source: JAMA Netw Open. 2025 Jan 13;8(1):e2453775. doi: 10.1001/jamanetworkopen.2024.53775 (PMC11731157; doi:10.1001/jamanetworkopen.2024.53775)
Supplement: Supplement 2. — Data Sharing Statement [file jamanetwopen-e2453775-s002.pdf]

Data Sharing Statement

Allis. Perceptions of Clinical Connectedness Among Hospital Environmental Service Workers. *JAMA Netw Open*. Published January 13, 2025. doi:10.1001/jamanetworkopen.2024.53775

Data
Data available: No
